# High genetic similarity of ciprofloxacin-resistant *Campylobacter jejuni* in central Europe

**DOI:** 10.3389/fmicb.2015.01169

**Published:** 2015-10-23

**Authors:** Jasna Kovač, Neža Čadež, Beatrix Stessl, Kerstin Stingl, Igor Gruntar, Matjaž Ocepek, Marija Trkov, Martin Wagner, Sonja Smole Možina

**Affiliations:** ^1^Department of Food Science and Technology, Biotechnical Faculty, University of LjubljanaLjubljana, Slovenia; ^2^Department for Farm Animals and Veterinary Public Health, Institute of Milk Hygiene, Milk Technology and Food Science, University of Veterinary MedicineVienna, Austria; ^3^National Reference Laboratory for Campylobacter, Department of Biological Safety, Federal Institute for Risk AssessmentBerlin, Germany; ^4^Institute of Microbiology and Parasitology, Veterinary Faculty, University of LjubljanaLjubljana, Slovenia; ^5^Department for Public Health Microbiology Ljubljana, Centre for Medical Microbiology, National Laboratory of Health, Environment and FoodLjubljana, Slovenia

**Keywords:** *Campylobacter jejuni*, ciprofloxacin resistance, multilocus sequence typing, *gyrA*, QRDR, resistance expansion

## Abstract

Campylobacteriosis is the leading zoonosis in the European Union with the majority of cases attributed to *Campylobacter jejuni.* Although the disease is usually self-limiting, some severe cases need to be treated with antibiotics, primarily macrolides and quinolones. However, the resistance to the latter is reaching alarming levels in most of the EU countries. To shed light on the expansion of antibiotic resistance in central Europe, we have investigated genetic similarity across 178 ciprofloxacin-resistant *C. jejuni* mostly isolated in Slovenia, Austria and Germany. We performed comparative genetic similarity analyses using allelic types of seven multilocus sequence typing housekeeping genes, and single nucleotide polymorphisms of a quinolone resistance determining region located within the DNA gyrase subunit A gene. This analysis revealed high genetic similarity of isolates from clonal complex ST-21 that carry *gyrA* allelic type 1 in all three of these central-European countries, suggesting these ciprofloxacin resistant isolates arose from a recent common ancestor and are spread clonally.

## Introduction

Campylobacteriosis is the leading zoonosis in the European Union (EU), with the majority of cases attributed to *Campylobacter jejuni* (EFSA, 2015). Severe or prolonged cases of campylobacteriosis are conventionally treated with antibiotics from the macrolide (erythromycin) and quinolone (ciprofloxacin) classes. While macrolide resistance is successfully retained at low levels (human isolates EU average, 1.5%), some EU Member States report up to 91.5% prevalence of quinolone resistance (EFSA, 2015). The resistance prevalence among *C. jejuni* from humans was 64.1 and 63.0% in Slovenia and Austria in 2013, respectively (EFSA, 2015). The main mechanism that confers high-level resistance to ciprofloxacin in *Campylobacter* is the occurrence of the Thre86Ile point mutation in the quinolone-resistance-determining region (QRDR) of the gene that encodes DNA gyrase subunit A (*gyrA*), although other mutations within *gyrA*, as well as enhanced eﬄux activity can contribute to development of ciprofloxacin resistance (Wieczorek and Osek, 2013). The Thre86Ile resistant mutants have also been demonstrated to have increased chicken colonization fitness (Luo et al., 2005) in certain genetic backgrounds, giving them an advantage over susceptible strains. In other genetic backgrounds, the advantage was not observed (Zeitouni and Kempf, 2011). The resistance to quinolones is especially problematic due to their therapeutic use in human and veterinary medicine, including in food production animals, which allows for transmission of antibiotic resistance along the food-production chain to humans (EMA/ESVAC, 2011).

Several studies have reported the resistance and genotype data that demonstrated increased prevalence of resistance in certain genotypes in different geographical regions (Kinana et al., 2006: D’lima et al., 2007; Gu et al., 2009; Habib et al., 2009; Wirz et al., 2010; Stone et al., 2013; Wu et al., 2014; Jonas et al., 2015). Furthermore, associations of particular multilocus sequence typing (MLST) clonal complexes with resistance were suggestive of clonal expansion of ciprofloxacin resistance in Slovenia, the UK and Switzerland (Cody et al., 2012; Kittl et al., 2013; Wimalarathna et al., 2013; Kovač et al., 2014).

To provide better insight into the epidemiology of *C. jejuni* antibiotic resistance in central Europe, we performed a comparative genetic similarity analysis of 178 ciprofloxacin-resistant *C. jejuni* strains isolated in Slovenia, Austria and Germany. We used standard MLST coupled with single nucleotide polymorphism (SNP) analysis of the QRDR of *gyrA* to investigate the genetic relatedness of ciprofloxacin resistant *C. jejuni* isolates and shed light on the nature of their expansion in these three central-European countries.

## Materials and Methods

### Bacterial Strains

The 178 ciprofloxacin resistant *C. jejuni* strains included in this study were isolated from poultry, cattle, chicken meat, cow milk, surface water, dog, strawberry, a zoo animal, and human stools in central Europe (Slovenia, Austria, Germany); two isolates were obtained from the southern Balkan region (Serbia, Bosnia, and Herzegovina) (**Supplementary Table S1**). The strains were isolated between 2001 and 2013 according to ISO10272, as part of national monitoring of retail meat and production animals, human clinical cases, and from other national and EU research projects.

### Bacterial Growth Conditions and DNA Extraction

Stocks of *C. jejuni* isolates were stored at -80°C and grown on selective Karmali agar (Oxoid, Hampsire, UK) for 24 h at 42°C under microaerobic conditions (5% O_2_, 10% CO_2_, in N_2_). DNA used for molecular species confirmation and MLST typing was extracted with PrepMan Ultra Sample Preparation Reagent (Applied Biosystems, Foster City, CA, USA), according to the manufacturer protocol. DNA was used for *C. jejuni* species confirmation according to Wang et al. (2002), and amplification of MLST housekeeping genes and *gyrA* QRDR. All primers used in this study are listed in **Supplementary Table S2**.

### Antibiotic Resistance

Resistances to ciprofloxacin (0.06–4 mg/l), chloramphenicol (2–32 mg/l), erythromycin (0.5–32 mg/l), gentamicin (0.12–16 mg/l), streptomycin (1–16 mg/l) and tetracycline (0.25–16 mg/l) were determined by broth microdilution using Eucamp microtitre plates (Sensititre, Thermo Fischer Scientific) by following manufacturers’ instructions. The reference strain ATCC 33560, which is susceptible to all seven tested antibiotics was used as a quality control. The minimum inhibitory concentration (MIC) cut-offs as recommended by EFSA (2015) were used to identify the resistant phenotypes. Briefly, isolates with MICs of ciprofloxacin >0.5 mg/l, chloramphenicol >16 mg/l, erythromycin >4 mg/l, gentamicin >2 mg/l, streptomycin >4 mg/l, and tetracycline >1 mg/l were identified as resistant. The MICs and resistance breakpoints are available in **Supplementary Table S1**.

### Multilocus Sequence Typing

**FIGURE 1:**
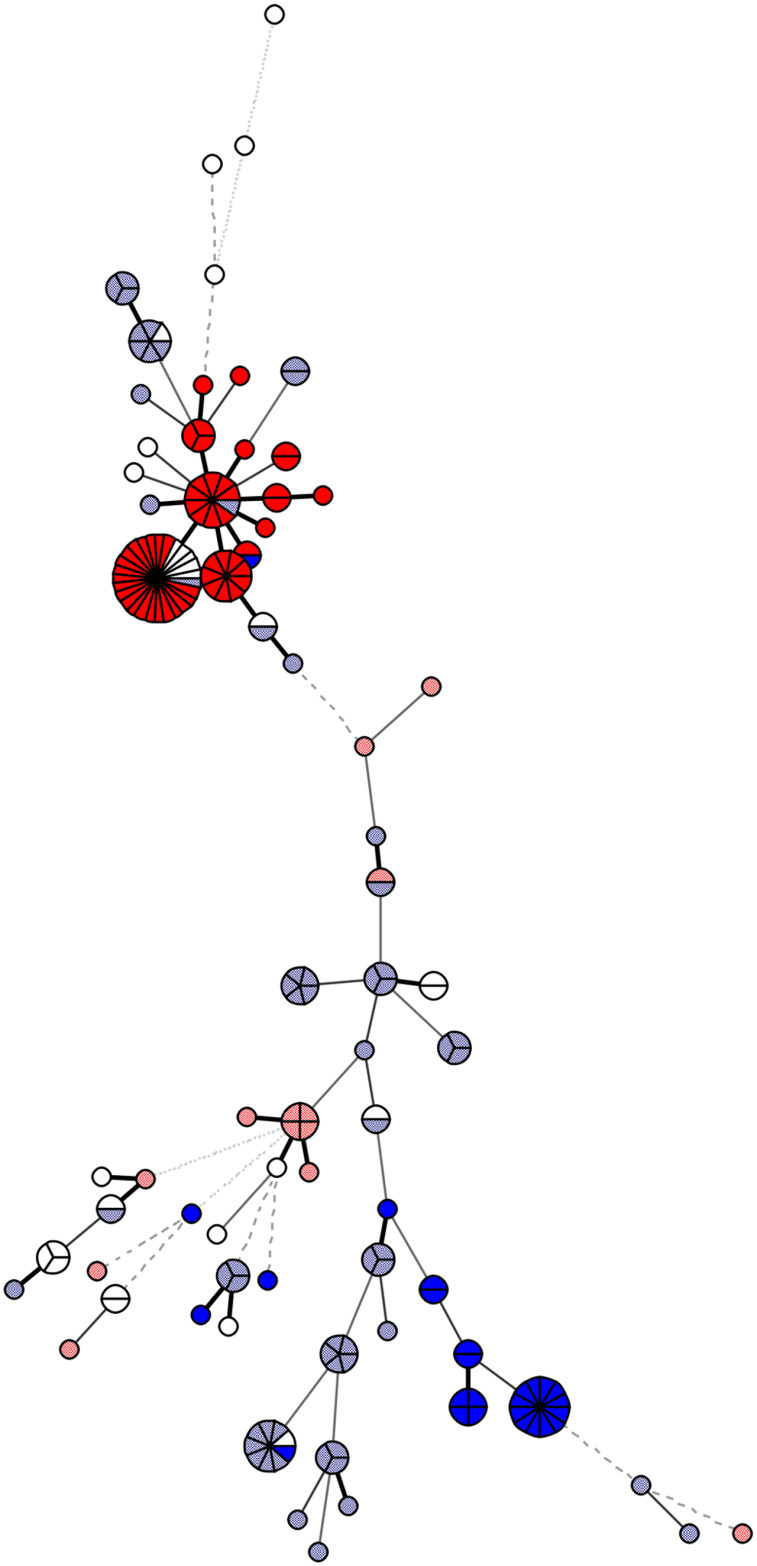
**Genetic similarity of the 178 analyzed *Campylobacter jejuni* isolates**. The minimum spanning tree was constructed based on the multilocus sequence typing (MLST) allelic profiles using Bionumerics, version 7.1. The genotype specific for clonal complex ST-21 + *gyrA* 1 is presented in dark red and for *gyrA* 1 in combination with a clonal complex other than ST-21 is in light red; the genotype for clonal complex ST-353 + *gyrA* 13 is in dark blue; and for *gyrA* 13 in combination with a clonal complex other than ST-353 is in light blue. The figure demonstrates higher similarity of isolates carrying *gyrA* allelic type 1, which cluster within clonal complex ST-21 (dark red), compared to isolates carrying *gyrA* 13, which are found within clonal complex ST-353 (dark blue), but are also dispersed among other clonal complexes (light blue).

Multilocus sequence typing was carried out according to the established *C. jejuni* MLST scheme, with primers listed in **Supplementary Table S2** (Dingle et al., 2001). Sequences (Sanger sequencing; Macrogen, South Korea) were used for the identification of MLST profiles using Bionumerics 7.1 with the MLST plugin (Applied Maths NV, Sint-Martens-Latem, Belgium). The MLST profiles identified in this study have been deposited in the PubMLST database http://pubmlst.org/campylobacter (Jolley and Maiden, 2010). The PubMLST IDs are available in **Supplementary Table S1**. The minimum-spanning tree (**Figure 1**) demonstrating the genetic similarity among strains was built using MLST allele variants in Bionumerics version 7.1 (Applied Maths).

### Quinolone-Resistance-Determining Region

The QRDR of *gyrA* of 178 ciprofloxacin-resistant *C. jejuni* isolates was amplified using primers GZgyrA5 and GZgyrA6 in a single PCR, and Sanger sequenced using primers GZgyrA7 and GZgyrA8 (Zirnstein et al., 1999; **Supplementary Table S2**). The 389-bp-long QRDR sequences spanning from nucleotide positions 92 to 481 within *gyrA* were aligned in MEGA V6.0 (Tamura et al., 2013) to identify the unique variants of *gyrA*. Every sequence with new SNP (synonymous or non-synonymous) observed was designated a new *gyrA* allelic type number. The conserved region of *gyrA* outside of QRDR was previously used by others for epidemiological investigation of *C. jejuni* and *C. coli* host specificity (Ragimbeau et al., 2014). Here we have investigated the QRDR region, as its sequence diversity is directly associated with ciprofloxacin resistance.

### Statistical Analysis

Statistical associations between *gyrA* types and MLST clonal complexes, and between *gyrA*+MLST types and multidrug resistance were carried out using Fischer’s exact tests in R, version 3.1.2, with the statistical relevance cut-off set at *p* < 0.01 (R Core Team, 2014).

## Results

**FIGURE 2:**
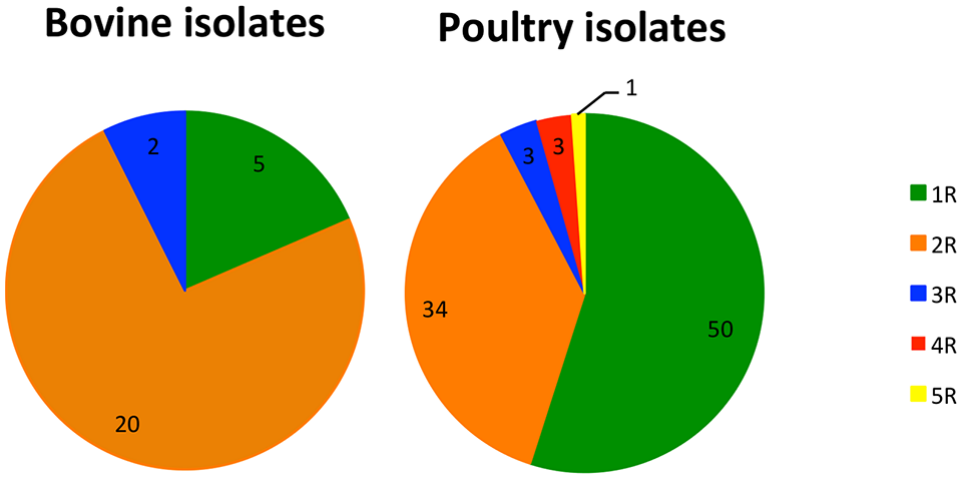
**Co-existence of quinolone resistance with resistances to other classes of antibiotics among isolates from bovine (*n* = 27) and poultry (*n* = 91) source**. 1R, 2R, 3R, 4R, 5R, resistance to quinolone alone (1R), and to antibiotics from one, two (exclusively quinolone and tetracycline), three, four and five other classes, respectively. Only isolates with resistance determined for all seven antibiotics tested ( *n* = 163) were included in the analysis.

Analyses of antibiotic co-resistance (multidrug resistance) were carried out on the isolates, with the MICs determined for all seven of the antibiotics tested (*n* = 163). The MICs of ciprofloxacin-resistant isolates ranged from 2 to over 4 mg/l, confirming a high-level resistance in all of them (resistance MIC cut-off is >0.5 mg/l). Among them, 76 (46.6%) isolates were either resistant to ciprofloxacin only (*n* = 4) or were cross-resistant to nalidixic acid (*n* = 72). The MICs of nalidixic acid ranged from 32 to over 46 mg/ml. Seventy-six isolates (46.6%) were co-resistant to ciprofloxacin and another antibiotic from a different class, which was exclusively tetracycline. MICs of tetracycline were ranging from 2 to over 16 mg/l, however most of the isolates (*n* = 61; 80.3%) were resistant to concentrations 16 mg/l or higher. Seven of the ciprofloxacin-resistant isolates (4.3%) were additionally resistant to either streptomycin and erythromycin, or streptomycin and tetracycline. Three isolates (1.8%) were resistant to antibiotics from four different classes, and a single isolate (0.6%) was resistant to antibiotics from five different classes (**Supplementary Table S1**). As demonstrated in **Figure 2**, higher proportion of resistance only to quinolone (1R) antibiotics was observed among isolates from poultry source (*n* = 50; 34.9%, *p* < 0.001), compared to isolates of bovine origin, where resistance to two classes of antibiotics (quinolones and tetracyclines – 2R) was prevailing (*n* = 20; 74.0%; *p* = 0.003). However, these differences arise mostly due to the high proportion of 1R among Slovenian poultry isolates (*n* = 33; 55%), compared to Austrian (*n* = 8; 17.4%) and German (*n* = 9; 33.3%), suggesting divergent antibiotic treatment practices in Slovenia, Austria, and Germany.

We identified 67 different MLST sequence types (STs) that were distributed among 21 clonal complexes (**Table 1**). Twenty isolates could not be assigned to any of the existing clonal complexes. Novel variants of alleles *aspA* (*aspA 387*), *glyA* (599), *tkt* (578), and *uncA* (458) were identified, along with the definition of their novel corresponding STs: ST 7514, ST 7517, ST 7516, and ST 7515, respectively. One new ST, ST 7506, was identified as a novel combination of previously reported allele types, according to the information in the PubMLST database.

**Table 1:** The distribution of ciprofloxacin-resistant *Campylobacter jejuni* isolates, originating from different countries among MLST clonal complexes.

	Austria	Germany	Slovenia	Serbia	Bosnia and Herzegovina	SUM
ST-21 complex	22	15	24			61
ST-353 complex	7	3	15		1	26
UA	10	1	9			20
ST-354 complex	2		8			10
ST-206 complex	5	1	3			9
ST-464 complex	6	1	1			8
ST-257 complex	4	1	1			6
ST-45 complex		4	2			6
ST-443 complex	4		1			5
ST-48 complex	2	1	2			5
ST-52 complex	1		4			5
ST-574 complex	4					4
ST-22 complex	1		1			2
ST-607 complex	1			1		2
ST-658 complex			2			2
ST-1034 complex		1				1
ST-283 complex			1			1
ST-362 complex			1			1
ST-42 complex		1				1
ST-49 complex	1					1
ST-692 complex		1				1
ST-828 complex			1			1
SUM	70	30	76	1	1	178

Sequence type 50 (clonal complex ST-21) was the most prevalent ST that was common to all three countries, Austria (*n* = 12), Slovenia (*n* = 12) and Germany (*n* = 3). The second-most frequent ST among the Slovenian isolates was ST 5205 (clonal complex ST-353) (*n* = 11), which was identified for the first time in our previous study (Kovač et al., 2014) and has been so far reported only for Slovenian (*n* = 11) and Austrian (*n* = 1) isolates, according to the PubMLST database (accessed on 20 June 2015). The most-prevalent clonal complex in all three of these countries was ST-21, with 24 representatives in Slovenia, 22 in Austria, and 15 in Germany, followed by ST-353, with 15 and seven representatives in Slovenia and Austria, respectively, and ST-45, with four representatives in Germany (**Supplementary Table S1**). Both, ST-21 and ST-353 were highly represented by chicken and human isolates, but no bovine isolate was assigned to ST-353. The 64.5% (*n* = 20; *p* < 0.001) of bovine isolates carried *gyrA* type 1, while 59.8% (*n* = 49, *p* < 0.001) of chicken isolates carried *gyrA* type 13.

The comparative analysis of the 389-bp-long QRDR sequences revealed 15 different *gyrA* allelic types with variations on 25 nucleotide sites (**Supplementary Table S1**; GenBank KP794628-KP794805). The sequences were checked for presence of previously reported resistance-conferring mutations on nucleotide positions 210, 255, 258, 270, and 312 (according to full-length *gyrA*), which corresponded to amino acids Ala70, Asp85, Thr86, Asp90, and Pro104 (Wieczorek and Osek, 2013). A total of 171 isolates carried the main resistance-conferring mutation Thr86Ile. This mutation was not detected in seven isolates, which still exhibited high level resistance to ciprofloxacin (MICs 2 to over 4 mg/l), possibly due to enhanced eﬄux activity. The *gyrA* types identified in isolates lacking the Thr86Ile mutation were *gyrA* 7, 8, 9, and 10. None of the isolates analyzed carried the alternative resistance-conferring mutations at amino-acid positions 70, 85, or 90, however five of the isolates had the Pro104Thr mutation. Pro104Thr is a newly identified mutation, as only Pro104Ser has been reported so far (Wieczorek and Osek, 2013). Pro104Thr was found in four strains that were isolated in Austria (08/000367, MRC-09/00280, MRC-10/00374, MRC-11/00224), and in a single strain isolated in Germany (Bfr-CA-10467) (**Supplementary Table S1**). All of the isolates with Pro104Thr mutation possessed the *gyrA* type 6.

The most prevalent *gyrA* STs were *gyrA* type 13 (*n* = 86) and *gyrA* type 1 (*n* = 65), with the remaining *gyrA* STs represented by only up to five isolates. The isolates with *gyrA* type 13 were distributed among 14 clonal complexes, and 26.7% of these isolates were assigned to ST-353 (ST-353 + *gyrA* 13; *p* < 0.001). On the other hand, isolates with *gyrA* type 1 were distributed among eight different clonal complexes, with 80.0% of these isolates clustering in ST-21 (ST-21 + *gyrA* 1; *p* < 0.001). Sequence analysis revealed closer relationships between isolates carrying *gyrA* type 1, which mostly belonged to clonal complex 21 (ST-21 + *gyrA* type 1), compared to isolates carrying *gyrA* type 13 (**Figure 1**). Both of these *gyrA* types were found in isolates from Slovenia, Austria, and Germany.

Isolates with genotype ST-353 + *gyrA* 13 were in most cases (*n* = 13; 61.9%; *p* < 0.001) resistant only to ciprofloxacin or to ciprofloxacin and nalidixic acid. Similar numbers, but lower proportions, of solely ciprofloxacin resistant isolates (*n* = 18; 39.1%; *p* = 0.2244) were observed also for genotype ST-21 + *gyrA* 1. A higher proportion of isolates with genotype ST-21 + *gyrA* 1 were resistant to two classes of antibiotics (*n* = 25; 54.3%; *p* = 0.222), than of isolates with genotype ST-353 + *gyrA* 13 (*n* = 5; 23.8%, *p* = 0.035). The proportions of isolates resistant to three antibiotics from different classes were low, both for ST-353 + *gyrA* 13 (*n* = 3; 14.3%) and for ST-21 + *gyrA* 1 (*n* = 1; 2.2%). Isolates that showed resistance to four and five antibiotics were represented only by genotype ST-21 + *gyrA* 1.

## Discussion

The current antibiotic-resistance problem among zoonotic agents demonstrates the need for improvements to resistance management policy. The EU ban on antibiotic use for growth promotion in animal husbandry was a step forward (European Commission, 2011). However, the extensive amounts of clinically important antibiotics that are applied for treatment and control of pathogens in food-production animals still support the spread and persistence of resistant bacterial populations in the food production chain, and therefore compromise efficient treatment of human infections (EMA/ESVAC, 2014).

Although the ban on the use of antibiotics in food animals may not produce the immediate targeted effects, studies from several countries that have established such policies have provided evidence for its sustainability, in terms of decreasing the prevalence of antibiotic resistance (Norström et al., 2006; Nelson et al., 2007; Skjøt-Rasmussen et al., 2009). Nevertheless, the implications of such policies and risk analyses for the expansion of antibiotic resistance through the international trade in chicken meat have not been evaluated to date, although such investigations are urgently needed.

Here, we have investigated the genetic similarity of ciprofloxacin-resistant strains isolated from the environment, animals, food and human in three central-European countries over the last 12 years to gain knowledge about their distribution among different genotypes that could potentially explain the mechanisms driving the increase in ciprofloxacin resistance prevalence in *C. jejuni*. We have found that resistance to ciprofloxacin alone (1R) is more frequently observed among poultry isolates, compared to bovine isolates where resistance to ciprofloxacin and tetracycline (2R) is more common. Although the associations were statistically significant according to Fischer’s exact test, the observations are biased, as substantially higher proportion of 1R isolates originates from Slovenia, compared to Austria and Germany. According to an opinion of Slovenian veterinary expert familiar with the field use of antibiotics in poultry, this can be explained with preferential use of enrofloxacin, rather than tetracyclines for poultry treatment in Slovenia. Main reason for that is the shorter withdrawal period and less problems with antibiotic precipitation in water.

Ciprofloxacin-resistant isolates clustered in two groups of high genetic similarity, as inferred based on MLST and SNP analyses of the QRDR. These two groups comprised isolates from MLST clonal complex ST-353 with *gyrA* type 13 and isolates from clonal complex ST-21 with *gyrA* type 1. While isolates from both of these MLST clonal complexes were significantly associated with their corresponding *gyrA* types, the sequence similarity analysis revealed closer relationships between isolates carrying *gyrA* type 1. Isolates with *gyrA* type 13 were widely dispersed also in clonal complexes other than ST-353, while isolates with *gyrA* type 1 belonged almost exclusively to MLST clonal complex 21, indicating they have expanded from a recent common ancestor. Furthermore, strains isolated from all three countries over several different years can be found within the group.

We have found 17 STs appearing between animal, as well as human isolates. Five of those (STs 50, 21, 104, 5205, and 572) were found more than once among human isolates. Of those, ST 50, 21, and 104 belong to clonal complex ST-21, ST 5205 to clonal complex ST-353, while 572 was unassigned. These findings suggest epidemiological linkage between animal and human isolates from clonal complexes ST-21 and ST-353, which also had the highest accumulation of ciprofloxacin resistant isolates.

The ST-21, and in particular ST 50 were highly represented among our set of ciprofloxacin resistant isolates, nevertheless previous studies in other geographical regions had not found significant associations between this genotype and quinolone resistance or susceptibility (Cody et al., 2012; Kittl et al., 2013). On the other hand, they have found ST 464 and ST 45 to be significantly associated with quinolone resistance, and susceptibility, respectively (Cody et al., 2012; Kittl et al., 2013). These two STs, however, were not identified frequently enough among our ciprofloxacin resistant isolates, to draw statistically relevant conclusions.

It is becoming increasingly evident that the global escalation of quinolone resistance in *Campylobacter* is associated with spreading of certain resistant genotypes (Cody et al., 2012; Kittl et al., 2013; Wimalarathna et al., 2013; Kovač et al., 2014), which may be driven by globalization in the poultry trade (AVEC, 2014) and/or of travel. Our findings suggest that the ciprofloxacin resistance is expanding with widespread *C. jejuni* clonal complex ST-21 in central Europe. Further research focused on identifying the genetic traits other than antibiotic resistance determinants that may facilitate this process is needed, in order to better understand the antibiotic resistance epidemiology, and implement successful resistance control measures.

## Conflict of Interest Statement

The authors declare that the research was conducted in the absence of any commercial or financial relationships that could be construed as a potential conflict of interest.

## Supplementary Material

TABLE S1 **Isolates used in this study**. The source of isolates, their antibiotic resistance profiles, MLST types and *gyrA* QRDR types.

TABLE S2 **Primers used in this study**.

## References

[B1] AVEC (2014). *Association of Poultry Processors and Poultry Trade in the EU Countries Annual Report.* Available at: http://www.avec-poultry.eu/system/files/archive/new-structure/avec/Annual\_Report/2014/Version\%20Finale.pdf

[B2] Cody A. J. McCarthy N. M. Wimalarathna H. L. Colles F. M. Clark L. Bowler I. C. (2012). A longitudinal 6-year study of the molecular epidemiology of clinical *Campylobacter* isolates in Oxfordshire, United Kingdom. *J. Clin. Microbiol.* 50 3193–3201. 10.1128/JCM.01086-12 22814466 PMC3457434

[B3] Dingle K. E. Colles F. M. Wareing D. R. Ure R. Fox A. J. Bolton F. E. (2001). Multilocus sequence typing system for *Campylobacter jejuni*. *J. Clin. Microbiol.* 39 14–23. 10.1128/JCM.39.1.14-23.2001 11136741 PMC87672

[B4] D’lima C. B. Miller W. G. Mandrell R. E. Wright S. L. Siletzky R. M. Carver D. K. (2007). Clonal population structure and specific genotypes of multidrug-resistant *Campylobacter coli* from turkeys. *Appl. Environ. Microbiol.* 73 2156–2164. 10.1128/AEM.02346-06 17293500 PMC1855654

[B5] EFSA (2015). The European union summary report on antimicrobial resistance in zoonotic and indicator bacteria from humans, animals and food in 2013. *EFSA J.* 13:4036 10.2903/j.efsa.2015.4036 PMC7009883 32625402

[B6] EMA/ESVAC (2011). *European Medicines Agency, European Surveillance of Veterinary Antimicrobial Consumption (ESVC). Trends in Sales of Veterinary Antimicrobial Agents in European Countires. Reporting Period 2005-2009.* Available at: http://www.ema.europa.eu/docs/en\_GB/document\_library/Report/2014/10/WC500175671.pdf [accessed April 13 2015].

[B7] EMA/ESVAC (2014). *European Medicines Agency, European Surveillance of Veterinary Antimicrobial Consumption, 2014. Sales of Veterinary Antimicrobial Agents in 26 EU/EEA Countries in 2012.* Available at : http://www.ema.europa.eu/docs/en_GB/document_library/Report/2014/10/WC500175671.pdf [accessed October 13 2015].

[B8] European Commission (2011). *Communication from the Commission to the European Parliament and the Council Action Plan Against the Rising Threats from Antimicrobial Resistance.* Available at: http://ec.europa.eu/dgs/health\_food-safety/docs/communication\_amr\_2011\_748\_en.pdf

[B9] Gu W. Siletzky R. M. Wright S. Islam M. Kathariou S. (2009). Antimicrobial susceptibility profiles and strain type diversity of *Campylobacter jejuni* isolates from turkeys in eastern North Carolina. *Appl. Environ. Microbiol.* 75 474–482. 10.1128/AEM.02012-08 19028914 PMC2620698

[B10] Habib I. Miller W. G. Uyttendaele M. Houf K. De Zutter L. (2009). Clonal population structure and antimicrobial resistance of *Campylobacter jejuni* in chicken meat from Belgium. *Appl. Environ. Microbiol.* 75 4264–4272. 10.1128/AEM.00168-09 19411429 PMC2704809

[B11] Jolley K. A. Maiden M. C. (2010). BIGSdb: scalable analysis of bacterial genome variation at the population level. *BMC Bioinformatics* 11:595 10.1186/1471-2105-11-595 PMC3004885 21143983

[B12] Jonas R. Kittl S. Overesch G. Kuhnert P. (2015). Genotypes and antibiotic resistance of bovine *Campylobacter* and their contribution to human Campylobacteriosis. *Epidemiol. Infect.* 143 2373–2380. 10.1017/S0950268814003410 25511436 PMC9150959

[B13] Kinana A. D. Cardinale E. Tall F. Bahsoun I. Sire J. M. Garin B. (2006). Genetic diversity and quinolone resistance in *Campylobacter jejuni* isolates from poultry in senegal. *Appl. Environ. Microbiol.* 72 3309–3313. 10.1128/AEM.72.5.3309-3313.2006 16672471 PMC1472360

[B14] Kittl S. Heckel G. Korczak B. M. Kuhnert P. (2013). Source attribution of human *Campylobacter* isolates by MLST and fla-typing and association of genotypes with quinolone resistance. *PLoS ONE* 14:e81796 10.1371/journal.pone.0081796 PMC3828285 24244747

[B15] Kovač J. Čadež N. Lušicky M. Nielsen E. M. Ocepek M. Raspor P. (2014). The evidence for clonal spreading of quinolone resistance with a particular clonal complex of *Campylobacter jejuni*. *Epidemiol. Infect.* 42 2595–2603. 10.1017/S0950268813003245 24534165 PMC9151304

[B16] Luo N. Pereira S. Sahin O. Lin J. Huang S. Michel L. (2005). Enhanced in vivo fitness of fluoroquinolone-resistant *Campylobacter jejuni* in the absence of antibiotic selection pressure. *Proc. Natl. Acad. Sci. U.S.A.* 102 541–546. 10.1073/pnas.0408966102 15634738 PMC545549

[B17] Nelson J. M. Chiller T. M. Powers J. H. Angulo F. J. (2007). Fluoroquinolone-resistant *Campylobacter* species and the withdrawal of fluoroquinolones from use in poultry: a public health success story. *Clin. Infect. Dis.* 44 977–980. 10.1086/512369 17342653

[B18] Norström M. Hofshagen M. Stavnes T. Schau J. Lassen J. Kruse H. (2006). Antimicrobial resistance in *Campylobacter jejuni* from humans and broilers in Norway. *Epidemiol. Infect.* 134 127–130. 10.1017/S0950268805004814 16409659 PMC2870368

[B19] R Core Team (2014). *R: A Language and Environment for Statistical Computing.* Vienna, R Foundation for Statistical Computing Avialble at: http://www.R-project.org/

[B20] Ragimbeau C. Colin S. Devaux A. Decruyenaere F. Cauchie H. Losch S. (2014). Investigating the host specificity of *Campylobacter jejuni* and *Campylobacter coli* by sequencing gyrase subunit A. *BMC Microbiol.* 14:205 10.1186/s12866-014-0205-7 PMC4156964 25163418

[B21] Skjøt-Rasmussen L. Ethelberg S. Emborg H. Agersø Y. Larsen L. S. Nordentoft S. (2009). Trends in occurrence of antimicrobial resistance in *Campylobacter jejuni* isolates from broiler chickens, broiler chicken meat, and human domestically acquired cases and travel associated cases in Denmark. *Int. J. Food. Microbiol.* 131 277–279. 10.1016/j.ijfoodmicro.2009.03.006 19345436

[B22] Stone D. Davis M. Baker K. Besser T. Roopnarine R. Sharma R. (2013). MLST genotypes and antibiotic resistance of *Campylobacter* spp. isolated from poultry in Grenada. *Biomed. Res. Internat.* 2013:794643 10.1155/2013/794643 PMC3595693 23555097

[B23] Tamura K. Stecher G. Peterson D. Filipski A. Kumar S. (2013). MEGA6: molecular evolutionary genetics analysis version 6.0. *Mol. Biol. Evol.* 30 2725–2729. 10.1093/molbev/mst197 24132122 PMC3840312

[B24] Wang G. Clark C. G. Taylor T. M. Pucknell C. Barton C. Price L. (2002). Colony multiplex PCR assay for identification and differentiation of *Campylobacter jejuni*, *C. coli*, *C. lari*, *C. upsaliensis*, and *C. fetus* subsp. fetus. *J. Clin. Microbiol.* 40 4744–4747. 10.1128/JCM.40.12.4744-4747.2002 12454184 PMC154608

[B25] Wieczorek K. Osek J. (2013). Antimicrobial resistance mechanisms among *Campylobacter*. *Biomed. Res. Int.* 2013:340605 10.1155/2013/340605 PMC3707206 23865047

[B26] Wimalarathna H. M. Richardson J. F. Lawson A. J. Elson R. Meldrum R. Little C. L. (2013). Widespread acquisition of antimicrobial resistance among *Campylobacter* isolates from UK retail poultry and evidence for clonal expansion of resistant lineages. *BMC Microbiol.* 5:160 10.1186/1471-2180-13-160 PMC3717071 23855904

[B27] Wirz S. E. Overesch G. Kuhnert P. Korczak B. M. (2010). Genotype and antibiotic resistance analyses of *Campylobacter* isolates from ceca and carcasses of slaughtered broiler flocks. *Appl. Environ. Microbiol.* 76 6377–6386. 10.1128/AEM.00813-10 20709846 PMC2950462

[B28] Wu Z. Sippy R. Sahin O. Plummer P. Vidal A. Newell D. (2014). Genetic diversity and antimicrobial susceptibility of *Campylobacter jejuni* isolates associated with sheep abortion in the united states and Great Britain. *J. Clin. Microbiol.* 52 1853–1861. 10.1128/JCM.00355-14 24648552 PMC4042809

[B29] Zeitouni S. Kempf I. (2011). Fitness Cost of fluoroquinolone resistance in *Campylobacter coli* and *Campylobacter jejuni*. *Microb. Drug Resist.* 17 171–179. 10.1089/mdr.2010.0139 21388301

[B30] Zirnstein G. Li Y. Swaminathan B. Angulo F. (1999). Ciprofloxacin resistance in *Campylobacter jejuni* isolates: detection of gyrA resistance mutations by mismatch amplification mutation assay PCR and DNA sequence analysis. *J. Clin. Microbiol.* 37 3276–3280. 10488192 10.1128/jcm.37.10.3276-3280.1999 PMC85547

